# Mutant TP53 promotes invasion of lung cancer cells by regulating desmoglein 3

**DOI:** 10.1007/s00432-024-05778-3

**Published:** 2024-06-20

**Authors:** Yu Feng, Rulin Qian, Dong Cui, Jiaqiang Luan, Mingxing Xu, Ling Wang, Ruijie Li, Xiao Wu, Chaoying Chang

**Affiliations:** 1 Department of Thoracic Surgery, Henan Provincial Chest Hospital, Zhengzhou University, No. 1 Weiwu Road, Zhengzhou, 450000 People’s Republic of China; 2 Department of Clinical Laboratory, Henan Provincial Chest Hospital, Zhengzhou University, Zhengzhou, 450000 People’s Republic of China; 3 Department of Medical Oncology, Henan Provincial Chest Hospital, Zhengzhou University, Zhengzhou, 450000 People’s Republic of China

**Keywords:** Lung cancer, TP53, Molecular interaction, Invasion

## Abstract

**Purpose:**

Targeted therapies have markedly improved the prognosis of lung cancer patients; nevertheless, challenges persist, including limited beneficiary populations and the emergence of drug resistance. This study investigates the molecular mechanisms of mutant TP53 in lung cancer, aiming to contribute to novel strategies for targeted therapy.

**Methods:**

The TCGA database was employed to delineate the mutational landscape of TP53 in lung cancer patients. Differential gene expression between TP53-mutant and wild-type patients was analyzed, followed by functional enrichment. DSG3 protein expression in lung cancer patients was assessed using IHC, and its impact on prognosis was analyzed in the TCGA database. The influence of TP53 on the downstream gene DSG3 was investigated using qPCR, ChIP-qPCR, and luciferase reporter gene assays. Protein enrichment in the DSG3 promoter region was examined through IP-MS, and the regulatory role of the HIF1-α/TP53 complex on DSG3 was explored using Co-IP, luciferase assays, and ChIP-qPCR. Molecular interactions between TP53 (R273H) and HIF1-α were detected through immunoprecipitation and molecular docking. The effects and mechanisms of DSG3 on lung cancer phenotypes were assessed through WB, transwell, and wound healing assays.

**Results:**

TP53 mutations were present in 47.44% of patients, predominantly as missense mutations. DSG3 exhibited high expression in TP53-mutant lung cancer patients, and this elevated expression correlated with a poorer prognosis. TP53 interference led to a reduction in DSG3 mRNA expression, with TP53 mutant P53 enriching at the P2 site of the DSG3 promoter region, a recruitment facilitated by HIF1-α. The DBD region of TP53 (R273H) demonstrated interaction with HIF1-α. DSG3, activated through Ezrin phosphorylation, played a role in promoting invasion and metastasis.

**Conclusions:**

Mutant TP53 facilitates lung cancer cell invasion by modulating desmoglein 3.

**Graphical abstract:**

**Figure:**
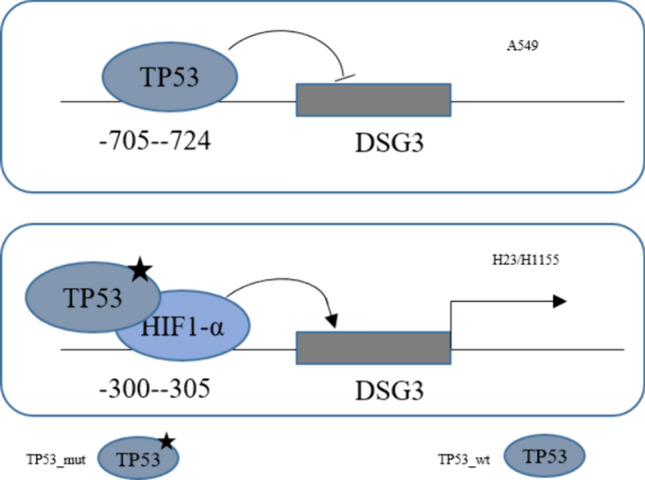
The regulation of TP53 on DSG3 in lung cancer cell

**Supplementary Information:**

The online version contains supplementary material available at 10.1007/s00432-024-05778-3.

## Introduction

The current epidemiological landscape of lung cancer encompasses its incidence and mortality rates, posing a formidable public health challenge (Duma et al. 2019). Despite therapeutic advancements, challenges persist in chemotherapy, underscoring the need for innovative approaches. Targeted therapies, exemplified by the targeting of EGFR mutations, have shown efficacy, yet their utility is limited by the low mutation rates and resistance issues in patients lacking EGFR mutations (Lahiri et al. 2023). Consequently, the imperative to identify novel targets for enhanced lung cancer treatment arises.

Desmoglein 3 (DSG3), a glycoprotein embedded in cell membranes and a member of the desmoglein family, is acknowledged for its pivotal role in mediating cellular adhesion within epithelial tissues (Louise and Hong 2015). Accumulating evidence implicates DSG3 in the progression of various cancer types, notably squamous cell carcinoma. (Florian et al. 2022) Hypoxia-inducible factor 1 alpha (HIF-1α) operates as a central orchestrator in cellular responses to hypoxia, steering adaptive mechanisms to maintain cellular equilibrium (Mohsen et al. 2021). HIF-1α engages in intricate interactions with various transcription factors, including c-Myc and p53 (Benjamin et al. 2023; Valentina et al. 2019), thereby regulating an array of cellular processes such as proliferation, programmed cell death, and metabolic activities.

The well-established tumor-suppressive function of wild-type (wt) TP53 is prominently acknowledged. Positioned on 17p13, the TP53 tumor suppressor gene spans a genomic region of 20 kilobases, encompassing 11 exons and encoding a 53 kDa nuclear phosphoprotein (Hu et al. 2021; Sullivan et al. 2018). The TP53 gene has been implicated in myriad biological processes, including cell-cycle arrest, apoptosis, metabolism, DNA repair, and senescence, thereby underscoring its pivotal role in orchestrating diverse cellular functions and upholding genomic stability (Mantovani et al. 2019; Shahbandi et al. 2020; Mansur and Greaves 2023). Among the approximately 22,000 genes in the human genome, TP53 experiences the highest mutation rate (Hu et al. 2021; Martincorena and Campbell 2015; Kaur et al. 2018). In non-small cell lung cancer (NSCLC), somatic mutations and increased TP53 expression are prevalent, reported at frequencies of approximately 23% and 65%, respectively (Volckmar et al. 2019; Cancer Genome Atlas Research Network 2014). These TP53 mutations are detected in tumors with or without allele loss at 17p13, predominantly clustering within the DNA-binding domain, featuring prominent hotspot mutations such as R246I and R273H (Wong et al. 2007). Consequently, this investigation delves into the regulatory mechanisms of mutant TP53 in the context of lung cancer, with the aspiration to provide novel perspectives for the targeted treatment of pulmonary malignancies.

## Material and methods

### Cell culture

NCI-H1155 (R273H), NCI-H23 (R246I), A549 (TP53 wild type) and HEK293 were obtained from ATCC. The DMEM/F12 medium (Gibco) supplemented with 10% FBS (Gibco) and 1% Penicillin–Streptomycin (Beyotime, China) was employed for culturing the H1155 cell line, while DMEM medium was used for the remaining cells. Under controlled conditions, cells were incubated at 37 °C in an atmosphere containing 5% CO2. The chemical induction of HIF-1α was achieved through treatment with CoCl_2_ (Sigma). siRNA (Invitrogen) was employed for cell transfection, which was carried out using Lipofectamine 3000 (Invitrogen) according to the manufacturer’s instructions.

### Plasmid construction

Expression vectors featuring distinct sequences of the R273H and the wt p53 were derived from H1155 and A549 respectively, through amplification of fragments utilizing the primers detailed in Table 1. The incorporation into the pcDNA-FLAG-HA plasmid (Addgene, 52535) was executed via a T4 DNA ligase reaction (New England Biolabs) subsequent to the enzymatic treatment of fragments and vector employing XhoI and EcoRI restriction enzymes (New England Biolabs). Plasmid lentiviral sgRNA/dCas9 constructs were individually customized by IGEbio (Guangzhou, China). The plasmids expressing dCas9 protein were transiently transfected into cells using Lipofectamine 2000, adhering to the manufacturer’s instructions.

**Table 1:** Primers

	Forward	Reverse
p53 1–393	TAGACTCGAGGAGGAGCCGCAGTCAGATC	GGTGGAATTCTCAGTCTGAGTCAGGCCCTTC
p53 98–324	TAGACTCGAGTCCCAGAAAACCTACCAGGG	GGTGGAATTCTCAATCCAGTGGTTTCTTCTT
p53 300–393	TAGACTCGAGGGGAGCACTAAGCGAGCACTG	GGTGGAATTCTCAGTCTGAGTCAGGCCCTTC
DSG3	GCCGATTTCATGGAAAGTTCTGAAG	TGGTTCCTCCAGTGGAATGC
DSG3_P1	ACACTGACAAAGTGTAGTCCGGAC	CAAGGGTAGGTCTCACCAGAAA
DSG3_P2	TATCATTGCTTAACATCTACATTTTGC	GGACTACACTTTGTCAGTGTTGATGT
DSG3_P3	CAGTATAAAAGATGTCATTTGAGTATTATC	CATCCCATTAAGCAACTATGAATGT
DSG3_P4	TAAGCAAAGTAGCTAAAATGGGGC	CAAAATTACTCTGATAATACTCAAATGACAT
DSG3_P5	CCGGGAGGCAGAGGTTG	CAGAAATATTGAAAGAAAGGTTTGGTA
siHIF1-a	GAGCTCCCAATGTCGGAGTTTGGAA	
siP53	GAATGAGGCCTTAGAGTTA	
siDSG3	GCAGAGAAGGAGAAGATAACT	
sgDSG3_P2	CCGAGTATGTAACAGCAAGCAGG	

### Clinical sample collection

A retrospective collection of FFPE samples from 30 patients diagnosed with lung cancer was conducted based on NGS results. The cohort comprised 15 patients with wild-type TP53 and 15 patients with TP53 mutations, the latter confirmed by experienced pathologists. Patient clinical data was extracted from the laboratory management system.

### TCGA data analysis

The analysis of The Cancer Genome Atlas (TCGA) data commenced with the retrieval of RNA-sequencing expression profiles, genetic mutations, and corresponding clinical information specific to lung cancer from the TCGA dataset (https://portal.gdc.com). Utilizing the maftools package within the R software (Mayakonda et al. 2018; Bi et al. 2020), mutation data were downloaded and visually presented. Genes exhibiting a heightened mutational frequency among LUAD patients were highlighted in a histogram. Differential expression of mRNA was investigated using the limma package in R, where thresholds for significance were set at “Adjusted P < 0.05” and “Log2 (Fold Change) > 1 or Log2(Fold Change) <  − 1”. Subsequently, to elucidate the potential functions of identified targets, functional enrichment analysis was conducted. The ClusterProfiler package (version: 3.18.0) in R was employed for Gene Ontology (GO) function analysis and Kyoto Encyclopedia of Genes and Genomes (KEGG) pathway enrichment (Yu et al. 2012). Visualization was achieved through boxplots using the ggplot2 package and heatmaps using the pheatmap package in R. In the context of enrichment results, pathways with a significance level of p < 0.05 or FDR < 0.05, as denoted by an enrichment score exceeding − log10 (P) of 1.3, were deemed meaningful.

### IHC detection

The 4 mm-thick FFPE tissue section underwent a baking process at 60 °C for 1 h in a drying oven. Subsequent deparaffinization transpired in xylene, accompanied by rehydration via a series of decreasing ethanol concentrations. Antigen retrieval for the tissue sections was accomplished through a 30-min incubation in retrieval solution (citrate buffer, pH 6) at a temperature of 95 °C. Sequentially, to mitigate non-specific binding, the tissue sections underwent treatment with 3% hydrogen peroxide and serum-free protein block solution (Dako, X0909), preceding the addition of DSG3 (abcam, ab183743), which underwent an incubation period of 1 h at room temperature. Signal amplification was realized using a biotinylated-secondary antibody and streptavidin–horseradish peroxidase. Subsequently, the slides underwent counterstaining with hematoxylin and were mounted utilizing a glycerol-based mounting medium. Tissue sections were evaluated under optical microscopy for staining intensity (graded from 0 to 3 as negative, faint yellow, light brown, and dark brown) and the extent of positivity (scored from 1 to 4 as 0–25%, 26–50%, 51–75%, and 76–100%). Subsequently, scores were summed for comparison.

### PCR detection

The extraction of RNA was executed utilizing TRIzol reagent (Invitrogen, USA) in adherence to standardized protocols. Quantification of the extracted RNA was achieved through the utilization of a Nanodrop 2000 spectrophotometer. Reverse transcription was facilitated using the PrimeScriptTM RT reagent kit (TAKARA, China). Following this, qRT-PCR reactions were conducted using the TB GREEN SuperMix (TAKARA, China) for the analysis of mRNA expression levels. To guarantee the normalization of data, the internal standard control selected was GAPDH.

### Luciferase reporter assay

Promoter luciferase constructs were generated by introducing a series of DSG3 promoter regions, subsequently inserted into the pGL3 vector. The resulting pGL3 vector, along with the pRL-TK Renilla luciferase reporter plasmid (E2241, Promega, Madison, USA) as an internal control to standardize for transfection efficiency, was transfected into H1155 and H23 cells. Following a 48-h incubation period, cellular harvests were conducted, and the quantification of firefly and Renilla luciferase activities in the lysates was performed in accordance with the manufacturer’s protocol (E1980, Promega).

### Immunoprecipitation

Following a 4-h exposure to CoCl_2_ (800 µM; Sigma), endogenous proteins were extracted via Trizol and subsequently sonicated to achieve fragment lengths ranging from 200 to 300 bp. Immunoprecipitation was executed by incubating cells overnight at 4 °C with antibodies targeting p53 (Santa Cruz, sc-126), HIF1-α (CST, #3716), or Cas9 (Invitrogen, #MA5-23519), followed by a 1-h incubation at 4 °C with protein-G agarose beads (Santa Cruz). Following centrifugation to isolate the precipitate, the supernatant was employed for DNA extraction and subsequent experiments. The precipitate underwent further analysis through Coomassie Brilliant Blue staining, Western blotting (WB), or mass spectrometry, the latter conducted by an external company.

### Immunoblotting

The protein, sourced from either immunoprecipitation in the preceding step or whole protein extraction, underwent immunoblotting following established procedures. Primary antibodies employed in this study encompassed GAPDH (Abcam, ab8245), anti-HA (CST, 3724), anti-Flag (CST, 14793), p53 (Santa Cruz, sc-126), HIF1-α (CST, 3716), DSG3 (Abcam, ab183743), Ezrin (pThr567) (ab76247), or Ezrin (Abcam, ab40839), and were incubated at 4 °C overnight. Subsequently, membranes were subjected to incubation with an HRP-conjugated secondary antibody. Band visualization and quantification were achieved using the Novex® enhanced chemiluminescence HRP Chemiluminescent Substrate Reagent Kit (Invitrogen) and the ChemiDocTM System (Bio-Rad, Richmond, CA, USA). Quantitative analysis of grayscale values was conducted through ImageJ software.

### Chromatin immunoprecipitation (ChIP)

Conforming to the guidelines specified by the manufacturer (Cell Signaling Technology), the ChIP assays were meticulously executed utilizing the SimpleChIP® Plus Kit. The DNA immunoprecipitated during the aforementioned IPs underwent elution and subsequent enrichment. Quantitative assessment was derived as a percentage relative to the input DNA, applying the formula 2[Input Ct − Target Ct] × 100 (%).

### Molecular docking

The 3D structures of TP53 R273H (4IBS) and HIF1-α (4H6J) were ascertained from the Protein Data Bank (PDB) (Eldar et al. 2013; Cardoso et al. 2012). To conduct molecular modeling, the PyMOL Molecular Graphics System (version 0.99 Schrödinger, LLC) was employed to prepare the chains of 4IBS and 4H6J, with histidine residues protonated at pH 6.5 using the PDB2PQR server 6 (Greenidge et al. 2013). Subsequently, TP53-active sites were identified utilizing the Active Site prediction server (http://www.scfbio-iitd.res.in/dock/ActiveSite.jsp), and the most substantial active pocket was selected for docking. The docking of TP53 and HIF1-α proteins was executed via the ZDOCK Server (Pierce et al. 2014). Ultimately, the PyMOL software was utilized for analytical visualization of the docking results, incorporating the annotation of all hydrogen bonds and their corresponding residues.

### Transwell

Cells in conjunction with matrigel (BD Biosciences, USA) were seeded into the upper chamber. After 24 h of incubation, the cells that did not undergo migration or invasion were eliminated using a cotton swab, whereas the cells situated on the bottom of the chamber were fixed with methanol for a duration of 10 min and subsequently stained with 0.5% crystal violet. Following this, a Nikon inverted microscope (Japan) was employed to capture images of five randomly chosen fields for documentation.

### Wound healing

Cells were initially plated in 24-well plates and permitted to incubate for a duration of 24–48 h until achieving full growth. Subsequent to this incubation period, wounds were induced utilizing 200 µl micropipette tips. Following the wound induction, cells underwent two rinses with 500 µl of PBS. Each well was then supplemented with 500 µl of serum-free medium. Sequential imaging was performed at 6-h intervals throughout a 24-h timeframe. Post-imaging, the acquired images underwent analysis through the utilization of ImageJ software.

### Data statistics

Statistical analyses were performed using R (version 4.2.1) and GraphPad Prism 8. Survival data within the TCGA dataset were evaluated through Kaplan–Meier analysis. For quantitative analysis of qPCR detection, a two-tailed unpaired Student’s t test was applied. Kruskal–Wallis and Mann–Whitney test was underwent for categorical data. Each experiment was iterated three times to ensure robustness and reproducibility. Statistical significance was established at P < 0.05, aligning with conventional thresholds for determining significance in biomedical research.

## Results

### Expression of DSG3 in patients with TP53 mutations

**Fig. 1:**
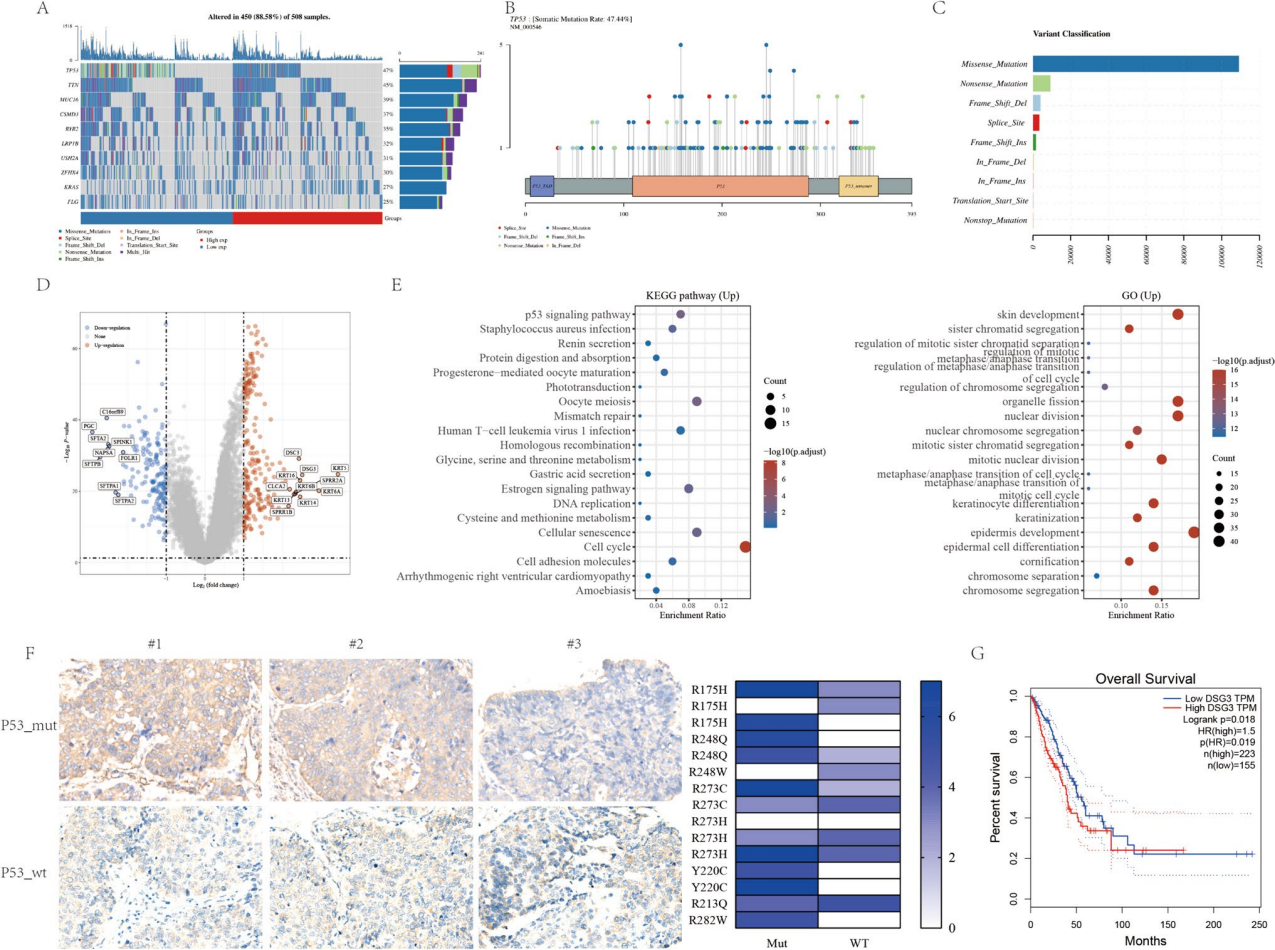
TP53 mutation in lung cancer patients and expression of DSG3. **A** Lollipop charts of mutated TP53 gene, figure caption displays somatic mutation rate, and subheadings indicate somatic mutation names. **B** Oncoplot illustrating somatic landscape of lung cancer cohort, genes ordered by mutation frequencies, samples ordered by disease histology, as annotated by the bottom bar. Sidebar plot presents −log10-transformed q values, estimated with MutSigCV. Waterfall plot depicts mutation details for each gene per sample. Color annotation for various cancer types displayed at the bottom. Barplot above the legend shows mutation burden count. **C** Cohort summary plot show cases variant distribution based on variant classification. **D** Volcano plot constructed using fold change values and P-adjust in 651 lung cancer patients with TP53 mutation and 350 lung cancer patients with wild-type TP53. Red dots signify upregulated genes; blue dots denote downregulated genes; grey dots indicate insignificance. **E** Functional enrichment: KEGG and GO analysis selected to illustrate primary biological actions of upregulated DEGs. Colors signify significance of differential enrichment, circle size denotes gene count, larger circles representing more genes. **F** IHC staining of DSG3 in lung cancer patients, photographed at 20× magnification. P = 0.009 using Mann–Whitney test between TP53_mut and TP53_wt group. **G** KM analysis of total survival period for lung cancer patients in TCGA database

A mutation analysis was conducted on 508 lung cancer patients sourced from TCGA, revealing TP53 as the most frequently mutated gene, with an incidence rate reaching 47.44% (Fig. 1A). Notably, these mutations predominantly manifested as missense mutations within TP53’s DNA-binding domain (DBD) (Fig. 1B, C). Simultaneously, an exploration of differentially expressed genes (DEGs) between TP53-mutated and wild-type lung cancer patients in TCGA data unveiled elevated transcription levels of DSG3 in the former (Fig. 1D). KEGG and GO analyses of these DEGs highlighted significant distinctions in signaling pathways such as cell adhesion molecules, epidermis development, and epidermal cell differentiation (Fig. 1E). Subsequent protein-level validation in clinical samples corroborated heightened DSG3 expression in TP53-mutated lung cancer patients (Fig. 1F). Furthermore, TCGA data underscored a correlation between elevated DSG3 expression and poorer prognosis in lung cancer patients (P = 0.018) (Fig. 1G). These findings collectively emphasize the potential prognostic relevance of DSG3 in TP53-mutated lung cancer.

### Binding of HIF1-α to the promoter region of DSG3 in P53-mutant cell lines rather than P53

**Fig. 2:**
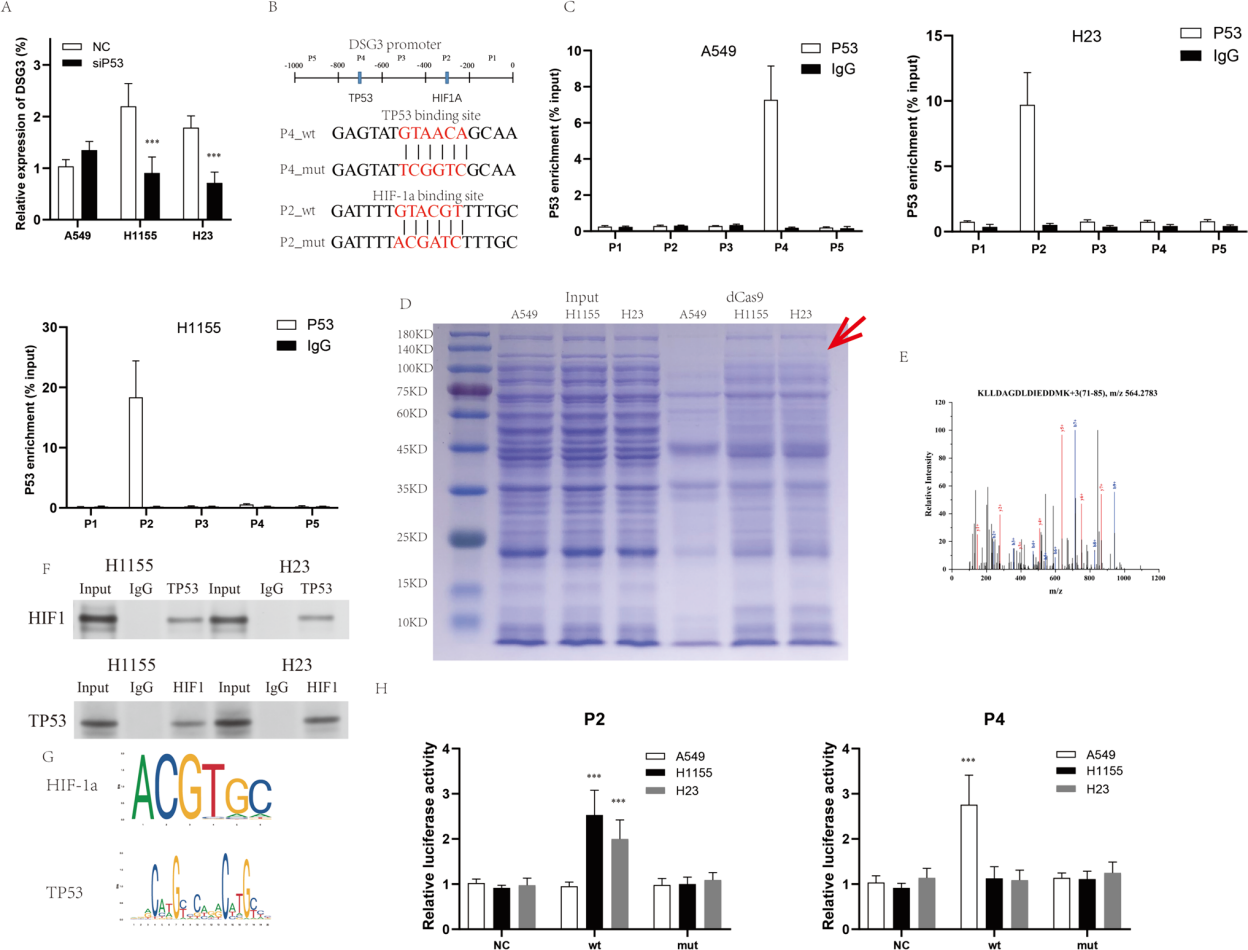
The role of TP53 and HIF1-α on DSG3. **A** The mRNA expression of DSG3 in lung cancer cells assessed following si-TP53. **B** A schematic representation of the structural of the DSG3 promoter region, along with diagrams illustrating two mutation sites. The upstream 1000 bp sequence of the promoter was divided into five segments of 200 bp each, denoted as P1–P5. **C** Determination of P53 protein enrichment on the five segments of the sequence in lung cancer cell lines utilized ChIP-PCR. **D** Application of Coomassie Brilliant Blue staining followed dCas9 immunoprecipitation, with significantly differential bands indicated by red arrows. **E** Generation of representative secondary structure mass spectrometry plots for HIF1-α. Inset: Fragmentation patterns of b and y ions displaying sequence information, amino acid residue, m/z, and charge state of the proteins. **F** Co-IP utilized for assessing interaction between HIF1-α and TP53 cells treated with 250 μM CoCl2 for 4 h. **G** Schematic plot illustrating binding motif of p53 and HIF1-α on DSG3 promoter. **H** Luciferase reporter gene assays conducted to measure luciferase activity at P2 and P4 sites

Widely acknowledged for its role as a transcription factor regulating target gene expression, TP53 was investigated for putative direct regulatory interactions with DSG3. Following TP53 interference, a reduction in DSG mRNA expression in mutant strains and a mild increase in DSG3 expression in wild-type strains were observed, indicative of TP53’s regulatory impact on DSG3 (Fig. 2A). Employing chip-pcr to examine five sites within the DSG promoter region, labeled P1–P5 (Fig. 2B), revealed TP53 enrichment at P4 in wild-type strains and at P2 in mutant strains (Fig. 2C). The differential TP53 binding sites between the two could be attributed to alterations in TP53 conformation. Literature reports suggest that mutant TP53 loses DNA binding capability (Kennedy and Lowe 2022; Yamamoto and Iwakuma 2018). Intriguingly, mutant TP53 binds to the P2 region, prompting discussion on potential recruitment by other transcription factors. Targeting the P2 site with gRNA and utilizing dcas9 antibody immunoprecipitation against three lung cancer cell lines, specific bands for 120KD were observed in mutant TP53 cell lines relative to A549 (Fig. 2D). Mass spectrometry identified HIF-1α as the most confidently associated proteins (Fig. 2E), validated by Co-IP demonstrating in vitro binding between HIF1-α and mutant TP53 (Fig. 2F). According to JASPR database predictions, the DSG3 promoter region P4 site (− 705- − 724) constitutes a TP53 binding site, while the P2 site (− 300- − 305) harbors an HIF1-α binding region (Fig. 2G). Fluorescent luciferase assays revealed a significant increase in activity upon transfection with wild-type P2 in H1155 and H23 cell lines, with minimal variation in mutant types, whereas A549 cells exhibited significantly enhanced fluorescence with wild-type P4 transfection (Fig. 2H), corroborating HIF1-α’s specific binding to the P2 site.

### Binding patterns of HIF1-α with mutant TP53

**Fig. 3:**
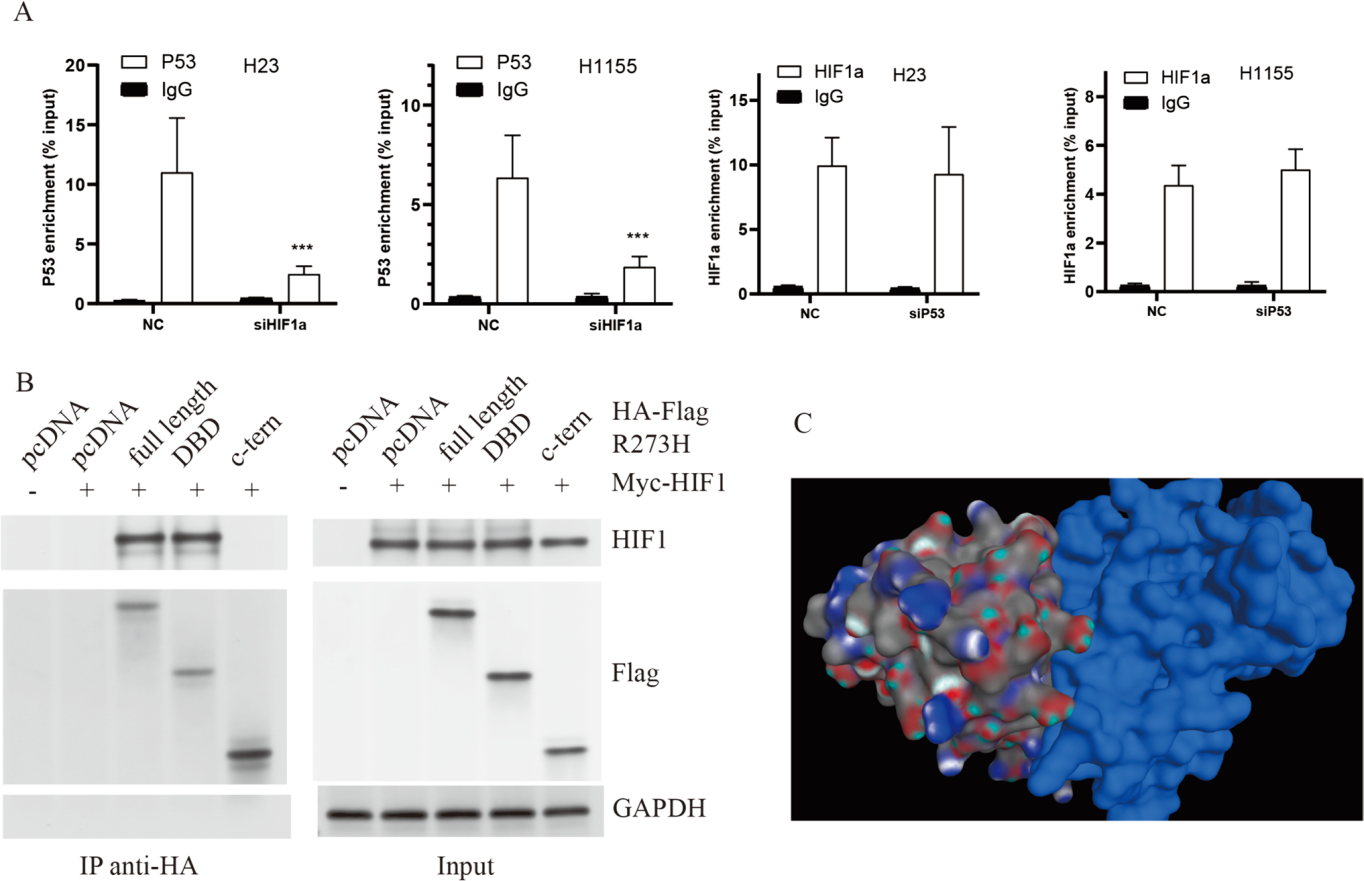
The interaction between mutant TP53 and HIF-1α. **A** The impact of TP53 and HIF-1α enrichment on the TP53 locus in the DSG3 promoter assessed through ChIP-PCR analysis. **B** HEK293 cells underwent Co-IP assays. Cells were subjected to 800 μm CoCl_2_ treatment and expressing myc-HIF-1α alongside various fragments of HA-flag–tagged TP53 R273H. **C** The binding interface of TP53 and HIF-1α was visually represented, with p53 depicted in grey and Hif1-α in blue

Upon the ablation of HIF1-α, a substantial reduction in TP53 enrichment at the DSG3 promoter was observed. Conversely, upon TP53 knockout, alterations in HIF1-α enrichment at the DSG3 promoter were not statistically significant, suggesting a pivotal role for HIF1-α in the regulation of DSG3 in wild-type TP53 cells (Fig. 3A). Subsequently, fragments overexpressing full-length HA-p53 R273H or Myc–HIF-1α were introduced into HEK293 cells. Co-IP analyses clearly delineated the involvement of the DNA-binding domain (DBD) of p53 R273H and HIF-1α in their physical interaction (Fig. 3B). Molecular docking between TP53 R273H and HIF1-α, facilitated by ZDOCK software, identified molecular conformations with the top 10 docking scores, as presented in Table S1. Visual analysis employing PyMOL software focused on the top-ranking molecular conformation (Fig. 3C). These findings conclusively elucidate the molecular interplay between TP53 and HIF1-α on the cellular surface of TP53 mutant cells.

### Promotion of DSG3 on invasion and migration in lung cancer cells

**Fig. 4:**
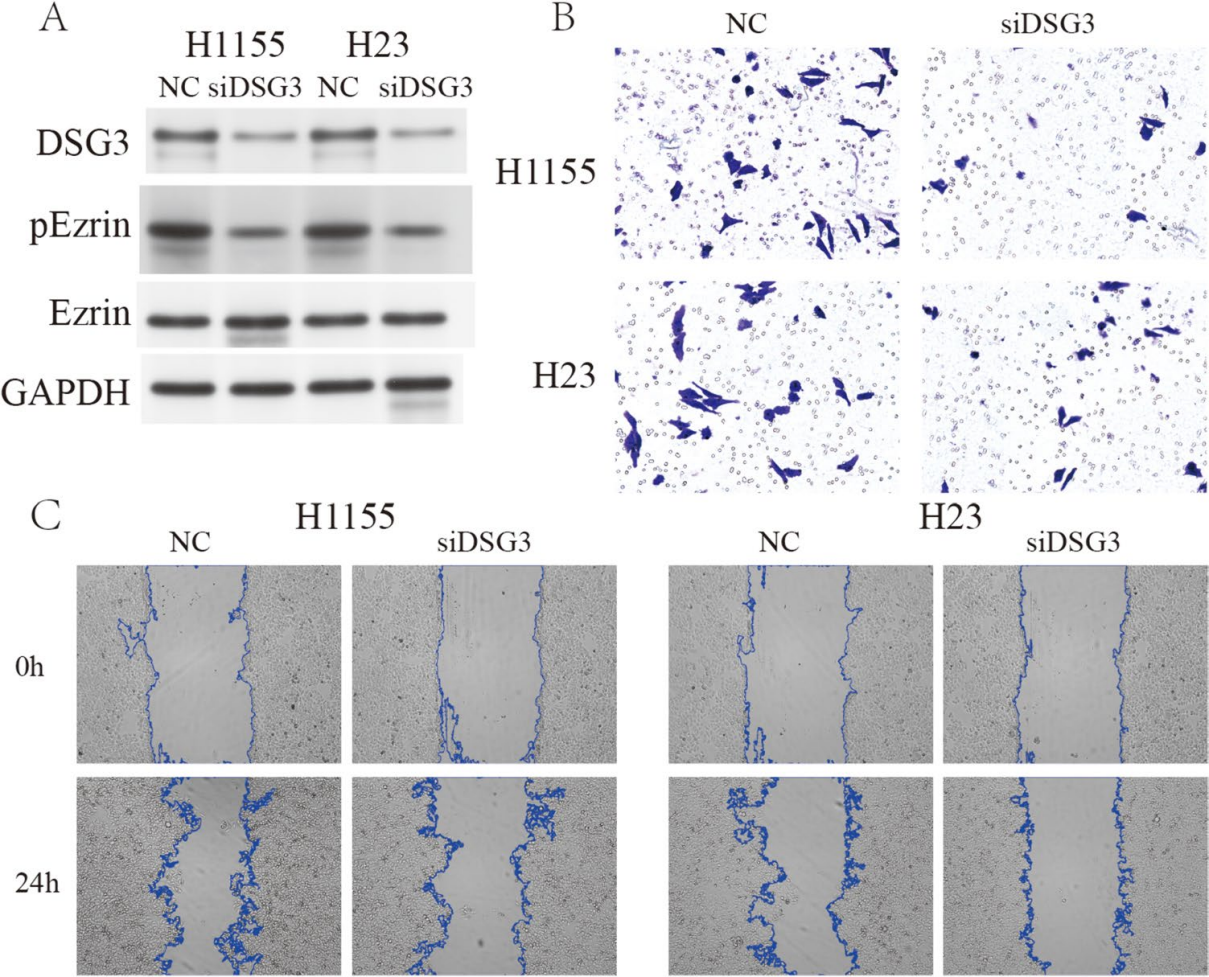
Impact of DSG3 on the invasive capability. **A** Activation of Ezrin facilitated by DSG3 through phosphorylation. **B** Evaluation of the invasive capacity of lung cancer cells performed through a transwell assay. **C** Assessment of cellular migration ability conducted utilizing a wound-healing assay

Reported evidence has indicated the facilitation of cancer cell migration and invasion through the regulation of Ezrin activation by DSG3 (23752190). In light of this, an investigation into the impact of DSG3 on the activation of Ezrin was undertaken. Following interference with DSG3, a notable reduction in the phosphorylation activation of Ezrin was observed, suggesting a potential modulatory role of DSG3 through the regulation of Ezrin phosphorylation (Fig. 4A). Furthermore, subsequent to DSG3 interference, a diminished migratory and invasive capacity was evident in H1155 and H23 cells (Fig. 4B, C). These findings substantiate the proposition that DSG3 influences cancer cell behavior, potentially through the modulation of Ezrin activation and, consequently, may serve as a critical player in cellular processes implicated in cancer progression.

## Discussion

In approximately half of human cancers, p53 undergoes direct inactivation through mutations. TP53 mutations can manifest as either structural mutants, altering the conformation of TP53 proteins, or contact mutants, affecting amino acids crucial for DNA binding (Walerych et al. 2015). Consequently, TP53 mutants lack the ability to transcriptionally activate wild-type TP53 target genes, rendering them unable to induce vital mediators of apoptosis, cell cycle arrest, cell senescence, and DNA damage repair. This phenomenon is commonly referred to as Loss of Function (LOF). However, not all observations can be attributed to LOF, as Mutant TP53 has been reported to exhibit neomorphic gain-of-function (GOF) properties (Freed-Pastor and Prives 2012), functions unattainable by wild-type TP53. Silencing mutant TP53 through siRNA has been demonstrated to impede the growth of specific tumor cells in vitro and in vivo, enhancing their susceptibility to cytotoxic drugs (Wang et al. 2022). The GOF effects of mutant TP53 have also been implicated in augmenting tumor metastasis by influencing transcription factors governing the epithelial-mesenchymal transition (EMT) (Ali et al. 2013; Roger et al. 2010). Moreover, mutant TP53’s GOF effects facilitate metabolic reprogramming in malignant cells to adapt to fluctuations in growth factors and nutrient availability, activating glycolysis (Zhang et al. 2013), promoting lipid synthesis (Freed-Pastor et al. 2012), and enhancing nucleotide synthesis (Kollareddy et al. 2015).

The dynamic interplay between wild-type (wt) members of the p53 family and Hypoxia-Inducible Factors (HIFs) has been extensively investigated, revealing a reciprocal regulatory relationship (Tiwari et al. 2020; Amelio et al. 2018). This intricate relationship gains particular significance in advanced cancers, including Non-Small Cell Lung Cancer (NSCLC), where TP53 gene mutations and HIF-1 activation in hypoxic regions are frequently observed (Amelio et al. 2018). Our investigations unveiled a distinctive mechanism wherein HIF1-α recruits mutant forms of TP53 within the DSG3 promoter region. This event leads to the loss of the tumor-suppressive nature inherent in wild-type TP53, activating DSG3 through a Gain-of-Function (GOF) mechanism. Consequently, this direct interaction instigates an invasive phenotype in lung cancer. The outcomes of our inquiry provide new insights into the molecular determinants that drive the aggressive characteristics of these tumors, specifically highlighting the direct interplay between mutant p53 forms and HIF-1 in NSCLC cells.

Formation of the TP53-HIF1-α complex has been extensively validated in numerous studies, as exemplified by instances where p53 mutants synergize with HIF-1 in the transcriptional regulation of extracellular matrix components, thereby fostering tumor progression (Amelio et al. 2018). Additionally, cooperative interactions between mutant p53 and the SWI/SNF chromatin remodeling complex have been reported, elucidating their regulatory role in VEGFR2 within breast cancer cells (Pfister et al. 2015). The TP53 protein delineates discrete functional domains, inclusive of the transactivation domain bifurcated into two N-terminal transactivation structures (TADI, 1–42, and TADII, 43–62), the proline-rich domain (PRD, 64–92), the central sequence-specific DNA-binding domain (DBD, 102–292), the oligomerization domain (OD, also termed the tetramerization domain, 323–356), and the C-terminal regulatory domain (363–393) (Sullivan et al. 2018; Kennedy and Lowe 2022). In our investigation, we observed the binding of the DNA-binding domain (DBD) of mutant TP53 to HIF1-α. This interaction culminates in the establishment of a transcriptional complex that governs the expression of DSG3, subsequently influencing the invasive phenotype of lung cancer cells through the Ezrin pathway. DSG3, a component of the desmosomal cadherins, is intricately involved in this process (Viehweger et al. 2022; Rehman et al. 2019). Simultaneously, Ezrin, belonging to the Ezrin/Radixin/Moesin (ERM) protein family, serves as a plasma membrane-actin linker and plays a pivotal role in cellular functions such as adhesion, polarization, and morphogenesis. Crucially, Ezrin emerges as a potent regulator of tumor cell invasion and metastasis (Barik et al. 2022; Qureshi-Baig et al. 2020; Li et al. 2019), emphasizing its multifaceted significance in cancer biology.

In summary, our findings provide insights into the molecular determinants potentially associated with lung cancer patients harboring mutant TP53. Moreover, these revelations emphasize prospective avenues for pioneering therapeutic strategies designed to mitigate the progression of lung cancer by selectively targeting the interplay between mutant p53 and HIF-1.

## Supplementary Information

Below is the link to the electronic supplementary material.

Supplementary Figure 1 (A) IHC staining of DSG1 in lung cancer patients, photographed at 20x magnification. (B) The expression of mRNA in lung cancer cell following si-TP53. (C) Detection of TP53 mRNA post-P53 interference (TIF 2468 KB)

Supplementary file2 (CSV 1975 KB)

## Data Availability

The data are available from the corresponding author on reasonable request.
