# Supplementary figures and images for: Mutant TP53 promotes invasion of lung cancer cells by regulating desmoglein 3
Source: J Cancer Res Clin Oncol. 2024 Jun 20;150(6):312. doi: 10.1007/s00432-024-05778-3 (PMC11189974; doi:10.1007/s00432-024-05778-3)

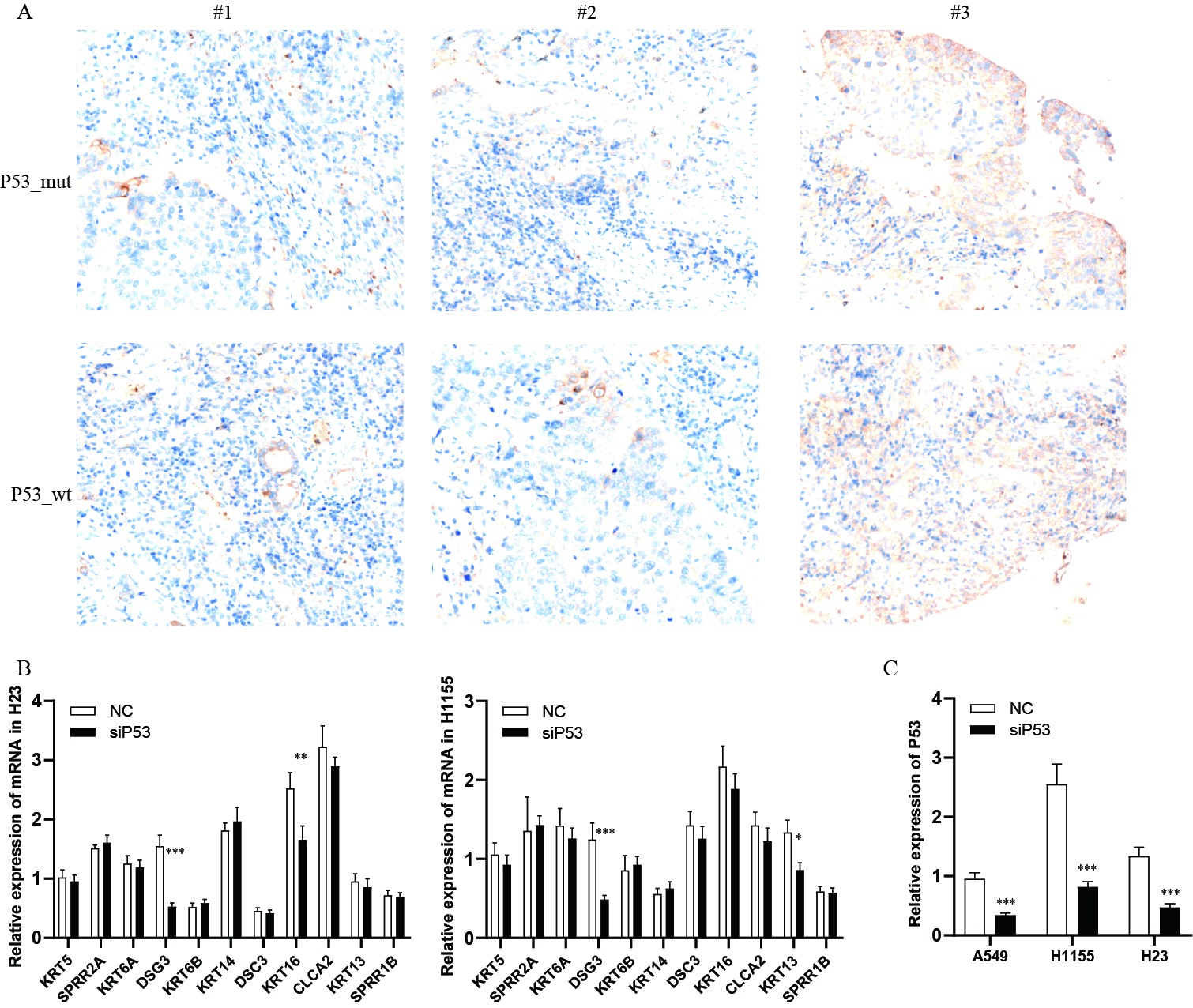

Supplement: Supplementary file 1 — Supplementary Figure 1 (A) IHC staining of DSG1 in lung cancer patients, photographed at 20x magnification. (B) The expression of mRNA in lung cancer cell following si-TP53. (C) Detection of TP53 mRNA post-P53 interference (TIF 2468 KB) [file 432_2024_5778_MOESM1_ESM.tif]
